# Leveraging Multiple Layers of Data To Predict *Drosophila* Complex Traits

**DOI:** 10.1534/g3.120.401847

**Published:** 2020-10-26

**Authors:** Fabio Morgante, Wen Huang, Peter Sørensen, Christian Maltecca, Trudy F. C. Mackay

**Affiliations:** * Department of Biological Sciences and W. M. Keck Center for Behavioral Biology, North Carolina State University, Raleigh, NC 27695; † Program in Genetics, North Carolina State University, Raleigh, NC 27695; ‡ Center of Quantitative Genetics and Genomics and Department of Molecular Biology and Genetics, Aarhus University, Tjele 8830, Denmark; § Department of Animal Science, North Carolina State University, Raleigh, NC 27695

**Keywords:** Genomic prediction, Transcriptomic prediction, Gene Ontology informed prediction, *Drosophila* Genetic Reference Panel, GenPred, Shared data resources

## Abstract

The ability to accurately predict complex trait phenotypes from genetic and genomic data are critical for the implementation of personalized medicine and precision agriculture; however, prediction accuracy for most complex traits is currently low. Here, we used data on whole genome sequences, deep RNA sequencing, and high quality phenotypes for three quantitative traits in the ∼200 inbred lines of the *Drosophila melanogaster* Genetic Reference Panel (DGRP) to compare the prediction accuracies of gene expression and genotypes for three complex traits. We found that expression levels (*r* = 0.28 and 0.38, for females and males, respectively) provided higher prediction accuracy than genotypes (*r* = 0.07 and 0.15, for females and males, respectively) for starvation resistance, similar prediction accuracy for chill coma recovery (null for both models and sexes), and lower prediction accuracy for startle response (*r* = 0.15 and 0.14 for female and male genotypes, respectively; and *r* = 0.12 and 0.11, for females and male transcripts, respectively). Models including both genotype and expression levels did not outperform the best single component model. However, accuracy increased considerably for all the three traits when we included gene ontology (GO) category as an additional layer of information for both genomic variants and transcripts. We found strongly predictive GO terms for each of the three traits, some of which had a clear plausible biological interpretation. For example, for starvation resistance in females, GO:0033500 (*r =* 0.39 for transcripts) and GO:0032870 (*r =* 0.40 for transcripts), have been implicated in carbohydrate homeostasis and cellular response to hormone stimulus (including the insulin receptor signaling pathway), respectively. In summary, this study shows that integrating different sources of information improved prediction accuracy and helped elucidate the genetic architecture of three *Drosophila* complex phenotypes.

Predicting complex traits is a fundamental aim of quantitative genetics. Historically, prediction of economically important traits has been important in animal and plant breeding, where the interest lies in predicting breeding values (*i.e.*, the additive genetic component of the phenotype) to select the best individuals for reproduction. Until recently, breeding values were predicted using mixed model methodology and pedigree relationships between individuals (Mrode and Thompson 2005). However, technological advances have enabled genotyping of individuals for tens to hundreds of thousands of single nucleotide polymorphisms (SNPs) throughout the entire genome. This has improved our ability to utilize information on genotypes and effects of SNPs on a trait of interest from individuals in a training population to predict breeding values for this trait as a linear combination of SNP genotypes for individuals in a target population which have genotype information only (Meuwissen *et al.* 2001; Goddard and Hayes 2009). The main advantages of the ‘genomic selection’ (GS) method over classical pedigree-based selection are the higher accuracy of the predicted breeding values due to capturing the Mendelian sampling variation, and the shortened generation interval since individuals can be genotyped as soon as they are born (Meuwissen *et al.* 2001; Schaeffer 2006; Hayes *et al.* 2009).

GS has been applied to many agricultural species and has revolutionized animal breeding; however, the results have varied greatly between species. Dairy cattle, which have the largest and highest quality reference populations, have the highest accuracy of genomic estimated breeding values. This high accuracy combined with the reduced generation interval has increased the rate of genetic gain up to a few orders of magnitude for many traits since the implementation of GS in breeding programs (Meuwissen *et al.* 2016; Weller *et al.* 2017). In beef cattle and pigs, where the reference populations are much smaller within each breed, the accuracy of genomic estimated breeding values is much lower. To overcome this issue, multi-breed reference populations have been used; however, across breed prediction also has low accuracy due to heterogeneous patterns of linkage disequilibrium (LD) across breeds (de Roos *et al.* 2009).

The GS framework can be more widely applied to non-pedigreed populations, where the goal is not to predict breeding values for the purpose of selection, but to predict individual phenotypes from genotype data, or ‘genomic prediction’ (GP). In human genetics, precision medicine refers to the goal of predicting the probability of developing a particular disease given an individual’s own genome (de los Campos *et al.* 2010; Goddard *et al.* 2016). Traditionally, human geneticists have used Polygenic Risk Scores (PRS) to predict complex traits. PRS are constructed as a weighted sum of the SNPs associated with the trait of interest from a genome wide association study (GWAS), with the estimated effects used as the weights (Dudbridge 2013; Wray *et al.* 2014). While PRS have had some significant predictive power, this has been generally limited (Machiela *et al.* 2011; Schizophrenia Working Group of the Psychiatric Genomics Consortium 2014). Another approach to GP is by regressing phenotypes on hundreds of variants concurrently using methods borrowed from animal breeding. This class of methods, called whole-genome regression (WGR), has the advantage of accounting for LD among SNPs when estimating their effects (de los Campos *et al.* 2010). However, early attempts at predicting human complex traits using WGR still yielded low prediction accuracy, especially in samples of unrelated individuals (Makowsky *et al.* 2011; de los Campos *et al.* 2013). Recently, much higher prediction accuracy was obtained using extremely large datasets combined with prediction methods that perform variable selection (Kim *et al.* 2017; Lello *et al.* 2018). These results highlight that adequate sample sizes and methods that account for trait architecture are needed to obtain higher prediction accuracy (Morgante *et al.* 2018).

In recent years, it has become possible to obtain multiple high quality ‘omic’ data (*e.g.*, gene expression levels, protein levels, metabolite levels) in addition to genotypes for the same samples. This has enabled integrating different types of data to uncover genotype-phenotype relationships using a systems genetics approach (Mackay *et al.* 2009; Ritchie *et al.* 2015). The first type of omic data to become available on a genome-wide scale were gene expression levels, initially measured by hybridization of genomic RNA to microarrays and now by direct RNA sequencing (RNA-seq). One way to use these data are to perform expression quantitative trait loci (eQTL) mapping to correlate expression levels with genetic variation (Gilad *et al.* 2008). Combining eQTL studies with GWAS revealed that significant hits for many diseases are likely to be eQTLs, meaning that disease-associated SNPs presumably act by altering gene expression levels (Nicolae *et al.* 2010). As other types of omic data became available, studies mapping protein QTL (pQTL) (Chick *et al.* 2016) and metabolic QTL (mQTL) (Kraus *et al.* 2015; Zhou *et al.* 2020) have helped elucidate how the effect of genetic variation percolates through intermediate molecular layers before affecting phenotypes.

A complementary approach utilizing multiple omics levels is for complex trait prediction, similar to prediction using genomic data. Different layers of data may provide (partially) non-redundant information about phenotypes (Guo *et al.* 2016). For example, gene expression levels may also capture environmental effects that affect levels of expression. In addition, gene expression traits, which are themselves genetically controlled, are much fewer and may have larger effects on phenotypes than genetic variants, resulting in an easier estimation problem.

Despite great promise, prediction of complex traits from multiple layers of omic data are in its infancy. Ehsani *et al.* (2012) used Bayesian methods to show that models including both genotypes and expression levels could achieve higher prediction accuracy than models with either one singularly. Wheeler *et al.* (2014) developed a method called OmicKriging that can incorporate different omics through similarity matrices, one for each omic level, among individuals. When applied to cell lines with RNA and micro-RNA data, a combined model including both types of omics achieved a higher prediction accuracy for a quantitative trait than the models including a single type of data. However, when applied to a clinical dataset for individuals with both DNA and RNA data, the combined model had lower prediction accuracy for a quantitative trait than the best single component model (Wheeler *et al.* 2014). Guo *et al.* (2016) used inbred lines of maize with genotype (G), gene expression level (T) and metabolite level (M) information to predict several complex traits using the Best Linear Unbiased Predictor (BLUP). In general, MBLUP yielded lower accuracy than all the other models. TBLUP and GBLUP provided similar accuracies, although, on average, GBLUP had better performance. In the majority of cases, poly-omic models (GTBLUP, GMBLUP, GTMBLUP) performed better than single-omic models. However, in many situations, the improvement provided by combined models was minimal (Guo *et al.* 2016). Vazquez *et al.* (2016) developed a Bayesian generalized additive model (BGAM) to incorporate multiple layers of omic data, each with a specific prior distribution. Using BGAM to integrate gene expression levels, DNA methylation levels and copy number variation status from The Cancer Genome Atlas generally improved accuracy of prediction of breast cancer survival in humans (Vazquez *et al.* 2016). Marigorta *et al.* (2017) developed transcriptional risk scores (TRS) using transcript abundance of genes with eQTLs that were in LD with or were inflammatory bowel disease (IBD)-associated SNPs. TRS outperformed PRS in distinguishing individuals with Crohn’s disease from controls, and TRS were able to predict disease progression whereas PRS were not (Marigorta *et al.* 2017). Li *et al.* (2019) used genotype, gene expression (obtained by tiling arrays; Huang *et al.* 2015), and phenotypic data from the *Drosophila melanogaster* Genetic Reference Panel (DGRP) (Mackay *et al.* 2012; Huang *et al.* 2014) to evaluate the proportion of variance explained and predictive ability of similar models to Guo *et al.* (2016). While models including expression data could capture a greater amount of variance, their predictive ability was generally similar to GBLUP (Li *et al.* 2019). Even more recently, two studies in *Drosophila* showed that using metabolites to predict a few complex traits can provide higher accuracy than genotypes (Zhou *et al.* 2020; Rohde *et al.* 2020).

These studies show that the use of multiple omics data to increase the prediction accuracy of complex traits is promising; however, the results are not consistent and are trait-specific. Here, we further examined the accuracy of poly-omic prediction of complex traits using 200 inbred, fully sequenced DGRP lines for which gene expression levels were recently obtained by RNA-seq (Everett *et al.* 2020). Taking advantage of the optimal experimental design, high quality omic data, and precise phenotype measurements, we sought to evaluate the prediction performance of either single-omic or poly-omic models using starvation resistance, startle response and chill coma recovery as model complex traits.

## Materials and Methods

### DGRP lines, genomic, transcriptomic and phenotypic data

The DGRP is a collection of 205 inbred lines derived from 20 generations of full-sib mating from isofemale lines collected at the Farmer’s Market in Raleigh, NC, USA. These lines were fully sequenced using a Illumina sequencing (Mackay *et al.* 2012; Huang *et al.* 2014). After retaining all the variants with minor allele frequency (MAF) > 0.05 and call rate > 0.8 for the 200 lines with gene expression levels, a total of 1,908,995 variants were used in the following analyses.

RNA-seq data have been obtained for 200 DGRP lines (Everett *et al.* 2020). RNA from whole young adult flies (3-5 days old) raised under standard conditions was extracted, rRNA depleted, sequenced using the Illumina HiSeq 2500 with 125 bp single-end reads. Two biological replicates each consisting of pooled groups of 30 flies were obtained for each of the two sexes. A total of 11,957 genes (10,251 known genes and 1,706 novel transcribed regions, NTRs) were genetically variable in females; and 13,672 genes (11,327 known genes and 2,345 NTRs) were genetically variable in males.

To obtain a list of genes that were highly expressed, we retained only the genes that had a mean expression across lines greater than −1.828 log_2_FPKM. This threshold was obtained from an analysis where a mixture model was fitted to the distribution of all expression values, which was bimodal (Everett *et al.* 2020). This procedure yielded 11,338 (9,807 known and 1,531 NTRs) and 13,575 (11,262 known and 2,313 NTRs) highly expressed genes in females and males, respectively. The following transcriptomic analyses were performed using only these genes.

The DGRP has been phenotyped for many complex traits (Mackay and Huang 2018). Here, we used line means for two fitness-related traits (starvation resistance and chill coma recovery time) and one behavioral trait (startle response) (Mackay *et al.* 2012). A total of 198, 172 and 199 lines for starvation resistance, chill coma recovery and startle response, respectively, had both phenotypic measurements and expression levels and were therefore retained for further analyses. The phenotypes were adjusted for the effects of *Wolbachia* infection and five major inversions (Huang *et al.* 2014).

### Whole genome and transcriptome prediction using linear mixed models

The data were analyzed using several different models. The baseline model was the Genomic Best Linear Unbiased Predictor (GBLUP). This is a linear mixed model where the covariance among lines is modeled using their realized relationships based on DNA marker loci (Habier *et al.* 2007). The model is y=1μ+y+e, where **y** is an *n*-vector of phenotypes, **1** is an *n*-vector of ones, *μ* is the population mean, **g** is an *n*-vector of random line genomic effects [**g** ∼ *N*(**0**, **G***σ*^2^*_g_*)] and **e** is an *n*-vector of random residual effects [**e** ∼ *N*(**0**, **I***σ*^2^*_e_*)]. **G** is the additive genomic relationship matrix (GRM) built using genetic variants according to the formula $\frac{WW^{’}}{p}$, where **W** is the matrix of centered and standardized genotypes for all the lines and *p* is the number of variants; **I** is the identity matrix.

To evaluate the performance of transcriptomic data for predicting complex traits, we used the Transcriptomic Best Linear Unbiased Predictor (TBLUP). This is very similar to GBLUP, but the GRM is substituted with a transcriptomic relationship matrix (TRM), which evaluates the similarity among lines based on gene expression levels (Guo *et al.* 2016). The model is y=1μ+t+e, where **y** is an *n*-vector of phenotypes, **1** is an *n*-vector of ones, *μ* is the population mean, **t** is an *n*-vector of random line transcriptomic effects [**t** ∼ *N*(**0**, **T***σ*^2^*_t_*)] and **e** is an *n*-vector of random residual effects [**e** ∼ *N*(**0**, **I***σ*^2^*_e_*)]. **T** is the additive TRM built according to the formula $\frac{ZZ^{’}}{m}$ where **Z** is the matrix of centered and standardized expression levels for all the lines and *m* is the number of genes; **I** is the identity matrix.

A combined model, GTBLUP, that had two variance components associated with the GRM and TRM, respectively, was also used. The model is y=1μ+g+t+e, where all the parameters are as defined above.

Finally, a fourth model, GTIBLUP, including three variance components associated with GRM, TRM, and the interaction of the two called IRM, respectively, was fitted. This model is y=1μ+g+t+g×t+e, where **y**, **1**, *μ*, **g**, **t** and **e** are as defined above, and **g**×**t** is an *n*-vector of random line interaction (between genomic and transcriptomic) effects [**g**×**t** ∼ *N*(**0**, **G**#**T***σ*^2^*_i_*) where # is the Hadamard product].

To avoid overfitting in the prediction analysis, 30 replicates of fivefold cross-validation were used; variance components were estimated in the training set using the ‘regress’ R package v. 1.3-15 (slightly modified by us to fix a small bug; available on the GitHub repository noted below), and then used to predict phenotypes in the test set. The proportion of variance explained in the training set by each component was calculated as the ratio of the variance explained by each component over the total phenotypic variance, estimated by REML (*e.g.*, in GBLUP, the proportion of variance explained by **g** is equal to $\frac{\sigma_{g}^{2}}{\sigma_{g}^{2}+\sigma_{e}^{2}}$), averaged over folds and replicates. Prediction accuracy in the test set was evaluated as the Pearson’s correlation coefficient, *r*, between true and predicted phenotypes, averaged over folds and replicates.

### Whole transcriptome prediction using Random Forest

Genes may affect traits, at least partially, through non-linear interactions. Therefore, we considered completely non-parametric methods that do not make the additivity assumption that linear mixed models do. Among the available methods, we chose the Random Forest (Breiman 2001), because it has been used for various bioinformatics tasks successfully, including the detection of interaction effects (Qi 2012; Yao *et al.* 2013). Following González-Recio and Forni (2011), the general Random Forest model can be written as $y=1\mu +\overset{S}{\underset{s=1}{\sum }}c_{s}h_{s}\left(y;X\right)$, where ***S*** is the number of decision trees, *c_s_* is a shrinkage factor that averages the trees, *h_s_(****y*; *X****)* is a single decision tree (independent of the others) grown on a bootstrap sample of the original data using only a subset of transcripts at each node, and ***X*** is the matrix of expression levels for all the lines. The algorithm implemented in the ‘randomForest’ R package v. 4.6.14 (Liaw and Wiener 2002) was fitted with default values of the tuning parameters and 1,000 trees. This analysis was performed using the same cross-validation scheme and metrics as the whole genome and transcriptome analysis.

### Transcriptome-Wide Association Study (TWAS) informed prediction

To try and enrich the TBLUP model for genes associated with the trait of interest and to eliminate noise from unassociated genes, variable selection was performed by repurposing the approach of Morgante *et al.* (2018). At each round of cross-validation, TWAS (regressing the phenotype on the expression level of each gene one at a time) was performed in the training set using the approach of Everett *et al.* (2020). The genes with a *p*-value < *X* (*X* = 0.5, 0.1, 10^−2^, 10^−3^, 10^−4^, 10^−5^, 10^−6^) were selected and used to build a trait-specific TRM. The trait-specific TRM was fitted in the TBLUP model to estimate variance components in the training set, to be used to predict phenotypes in the test set.

As a control, prediction using only randomly sampled genes was performed for the three traits. At each round of cross-validation, *k* genes (*k* = 5; 50; 500; 1,000; 5,000) were randomly sampled and used for prediction in a similar way to the TWAS-selected genes.

### Gene Ontology (GO) informed prediction

We used functional annotation, which relies on external sources of information, to further attempt to disentangle signal from noise. Edwards *et al.* (2016) showed that exploiting information about gene ontology categories could improve the accuracy of variant-based prediction. Here, we followed the same approach for variant-based prediction with our data and extended it to expression-based prediction. Variants were mapped to genes based on FlyBase v. 5.57 annotation (St Pierre *et al.* 2014). Genes were then mapped to Gene Ontology (GO) terms using the R package ‘org.Dm.eg.db’ v. 3.5.0 (Carlson 2017) available in BioConductor.

For SNP-based prediction, a GO-GBLUP model was fitted: $y=1\mu +g_{\mathrm{GO}}+g_{\mathrm{notGO}}+e$, where **g_GO_** is an *n*-vector of random line genomic effects associated with variants pertaining to a specific GO term (through a GO-specific GRM built using variants in a specific GO), **g_notGO_** is an *n*-vector of random line genomic effects associated with all the remaining variants (through a GRM built using all variants not in that GO), and all the other parameters are as defined above. This model was fitted for all GO terms including at least 5 genes, resulting in 2,605 GO terms.

For expression-based prediction, a GO-TBLUP model was fitted: $y=1\mu +t_{\mathrm{GO}}+t_{\mathrm{notGO}}+e$, where **t_GO_** is an *n*-vector of random line transcriptomic effects associated with genes pertaining to a specific GO term (through a GO-specific TRM built using genes in a specific GO), **t_notGO_** is an *n*-vector of random line transcriptomic effects associated with all the remaining genes (through a TRM built using all genes not in that GO), and all the other parameters are as defined above. This model was fitted for all GO terms including at least 5 genes that were present in our expression data, resulting in 2,346 and 2,287 GO terms for females and males, respectively.

In order to evaluate whether genome-level and transcriptome-level GO terms contribute overlapping information, the following combined model (GO-GTBLUP) was fitted: $y=1\mu +t_{\mathrm{GO}}+t_{\mathrm{notGO}}+g_{\mathrm{GO}}+g_{\mathrm{notGO}}+e$, where all the parameters are as defined above. This model was fitted for all GO terms that were in common between GO-variants and GO-genes after pruning according to the requirements described above, resulting in 2,338 and 2,282 GO terms for females and males, respectively.

All these analyses were performed using the same cross-validation scheme and metrics as the whole genome and transcriptome analysis. The mean proportion of variance explained by each GO term in the training data set was estimated as the ratio of the variance explained by the features in a specific GO term over the total phenotypic variance, averaged over folds and replicates (*e.g.*, in GO-GBLUP, the mean proportion of variance explained by **g_GO_** is equal to mean ($\frac{\sigma_{gGO}^{2}}{\sigma_{gGO}^{2}+\sigma_{gnotGO}^{2}+\sigma_{e}^{2}}$)).

All the statistical analyses were performed using Microsoft R Open v. 3.4.3 (https://mran.microsoft.com/).

### Data availability

All DGRP lines are available from the Bloomington Drosophila Stock Center (Bloomington, IN). All raw and processed RNA-Seq data are available at the NCBI Gene Expression Omnibus (GEO; https://www.ncbi.nlm.nih.gov/geo/) under accession number GSE117850. All quantitative trait data and genome sequence variant data are available at http://dgrp2.gnets.ncsu.edu. The code used for the analyses is available at https://github.com/morgantelab/multiomic-prediction-dgrp. Supplemental material available at figshare: https://doi.org/10.25387/g3.13133342.

## Results

We present the figures corresponding to all the analyses for starvation resistance in the main text and those for startle response and chill coma recovery are presented in the supplementary material.

### Variance partition using linear mixed models

**Figure 1:**
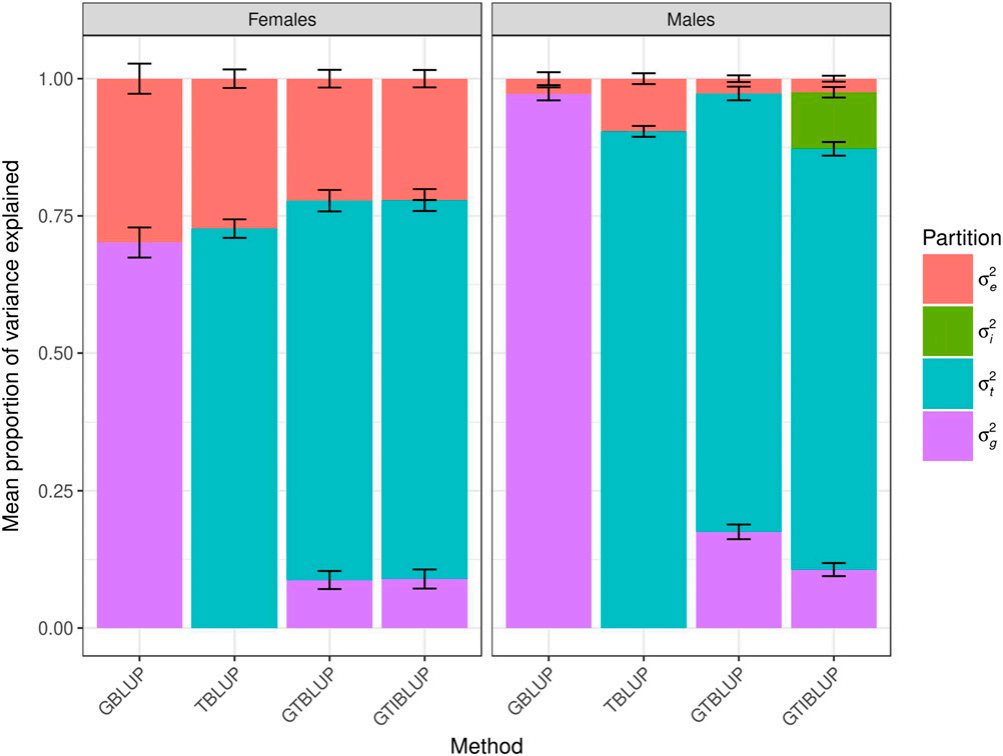
Mean proportion of phenotypic variance for starvation resistance explained by each component in the models fitted to the training data set, *i.e.*, GBLUP (*σ*^2^*_g_* and *σ*^2^*_e_*), TBLUP (*σ*^2^*_t_* and *σ*^2^*_e_*), GTBLUP (*σ*^2^*_g_*, *σ*^2^*_t_* and *σ*^2^*_e_*), and GTIBLUP (*σ*^2^*_g_*, *σ*^2^*_t_*, *σ*^2^*_i_* and *σ*^2^*_e_*). The bars represent the standard error of the mean. The left panel represents females, and the right panel represents males.

We first assessed to what extent the transcriptome could explain the phenotypic variance in the training data set for the traits of interest, compared to the variance explained by the genome, by fitting TBLUP and GBLUP models. Note that these analyses utilize line means of many individuals, so the majority of the phenotypic variance is expected to be genetic. GBLUP explained 70 ± 3% (97 ± 1%) of the phenotypic variability for starvation resistance in females (males). The performance of TBLUP was similar to GBLUP for this trait, explaining 73 ± 2% (90 ± 1%) of the phenotypic variability females (males) (Figure 1). However, this pattern did not hold for all three traits. For startle response, GBLUP explained 43 ± 2% (31 ± 2%) of the phenotypic variance in females (males) and TBLUP could only explain 8 ± 1% (18 ± 1%) of the trait variation in females (males) (Fig. S1). For chill coma recovery, GBLUP explained 19 ± 3% (11 ± 2%) of the phenotypic variance in females (males) while TBLUP was able to explain negligible phenotypic variance in either sex (Fig. S2).

We then fitted a joint genetic and transcriptomic model (GTBLUP) to evaluate the relative variance explained from the genome and the transcriptome. The performance of this model was relatively similar to TBLUP alone for starvation resistance in females, with the transcriptome explaining 69 ± 2% of the phenotypic variance and only 9 ± 2% being explained by the genome. For male starvation resistance, the model explained nearly all of the phenotypic variance, with 17 ± 1% of the variation explained by the genomic and 80 ± 1% by the transcriptome contributions (Figure 1). For startle response, the majority of the variance explained by GTBLUP was contributed by the genome, 39 ± 2%, while the transcriptome could only explain 5 ± 1% of the variance in females. However, the contributions of genome and transcriptome to variance of male startle response were more similar at 24 ± 2% and 13 ± 1%, respectively (Fig. S1). For chill coma recovery, GTBLUP gives similar results to GBLUP, with the genome explaining 20 ± 3% (12 ± 2%) and the transcriptome explaining negligible variance in females (males) (Fig. S2).

Finally, we fitted a model considering the genome, transcriptome and their interaction (GTIBLUP). For female starvation resistance, the performance of this model was very similar to that of GTBLUP. The transcriptome explained 69 ± 2%, the genome explained 9 ± 2% of the phenotypic variance, and negligible variance was explained by their interaction. For male starvation resistance, nearly all phenotypic variance was explained, with 11 ± 1% coming from the genome, 77 ± 1% from the transcriptome and 10 ± 1% from their interaction (Figure 1). For female startle response, the GTIBLUP model explained nearly half of the phenotypic variance, with 35 ± 2%, 5 ± 1%, and 7 ± 1% coming, respectively from the genome, transcriptome and their interaction. For male startle response, the GTIBLUP model was able to explain about a third of the phenotypic variance, 24 ± 2% from the genome and 12 ± 1% from the transcriptome, similar to GTBLUP; the interaction term explained negligible variance (Fig. S1). The GTIBLUP model explained 15% of the variance for chill coma recovery in males, with 10 ± 2% coming from the genome and 4 ± 1% from the interaction term; the transcriptome explained negligible variance. On the other hand, almost all the variance explained by GTIBLUP in female chill coma recovery came from the genome-transcriptome interaction (57 ± 2%) while the genome only explained 4 ± 1% of the variance, and negligible contribution from the transcriptome (Fig. S2).

### Whole genome and transcriptome prediction using linear mixed models

**Figure 2:**
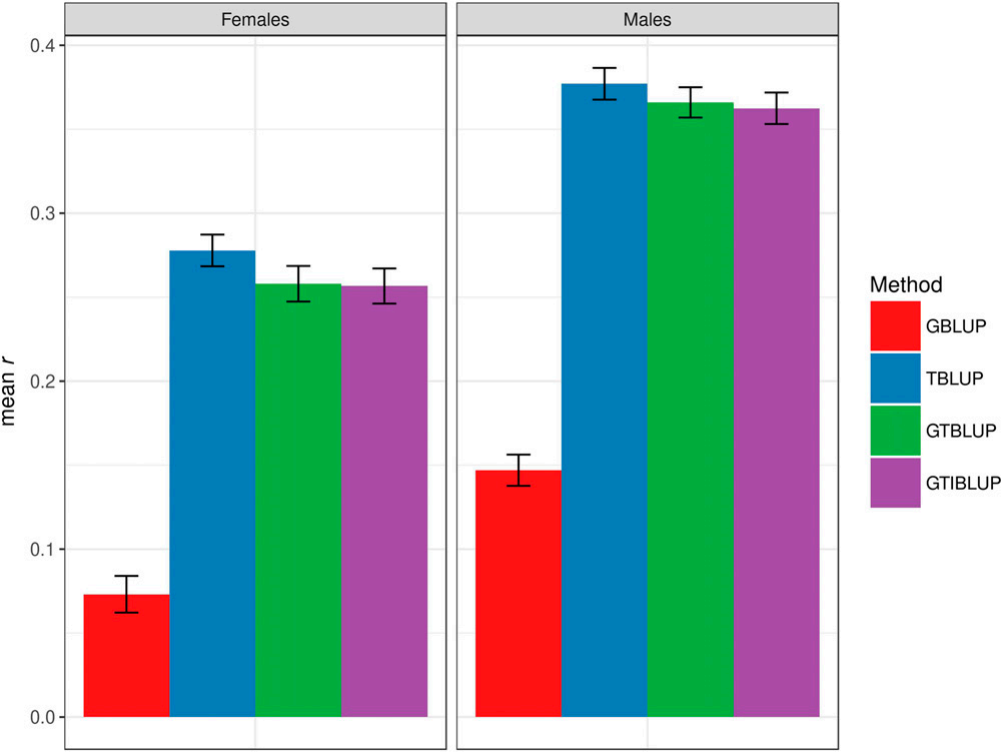
Prediction accuracy for starvation resistance, measured as mean *r* (on the y-axis) in the test set, obtained by GBLUP, TBLUP, GTBLUP, and GTIBLUP. The bars represent the standard error of the mean. The left panel represents females, and the right panel represents males.

Since the transcriptome contributes significantly to trait phenotypic variance in the training set in most cases, we next evaluated the predictive ability of the four models in the test set. For starvation resistance, the predictive abilities of each of the models was greater in males than females. The GBLUP model had the lowest prediction accuracy – *r* = 0.07 ± 0.01 (*r* = 0.15 ± 0.01) in females (males). The TBLUP model had greater than twice the prediction accuracy of GBLUP for starvation resistance, with *r* = 0.28 ± 0.01 (*r* = 0.38 ± 0.01) in females (males). The predictive abilities of the GTBLUP and GTIBLUP models were similar to TBLUP for starvation resistance (Figure 2). In contrast, the GBLUP model had the highest predictive ability for startle response, with *r* = 0.15 ± 0.01 (*r* = 0.14 ± 0.01) in females (males). The TBLUP model had prediction accuracies of *r* = 0.12 ± 0.01 and *r* = 0.11 ± 0.01 in females and males, respectively. The GTBLUP and GTIBLUP models has similar prediction accuracies to TBLUP in females (*r* = 0.10 ± 0.01 and *r* = 0.10 ± 0.01, respectively), whereas in males they had the lowest prediction accuracies (*r* = 0.08 ± 0.01 and *r* = 0.08 ± 0.01, respectively) (Fig. S3). Prediction accuracies for all four models for chill coma recovery were all less than zero (Fig. S4).

### Whole transcriptome prediction using Random Forest

**Figure 3:**
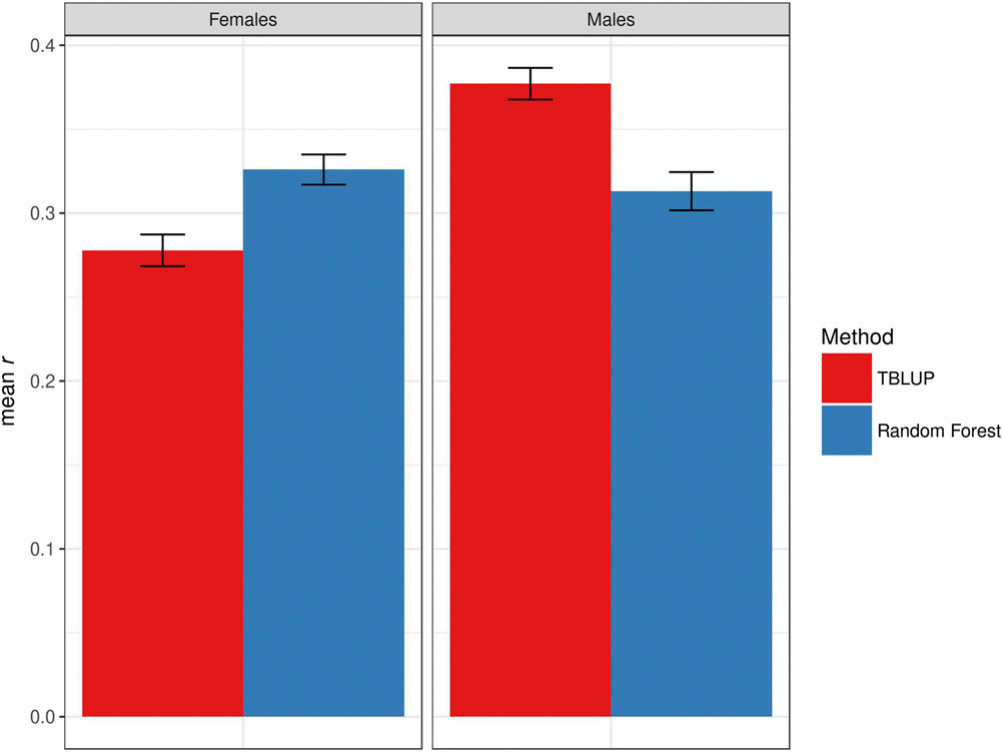
Comparison between the prediction accuracy for starvation resistance, measured as mean *r* (on the y-axis) in the test set, obtained by TBLUP and Random Forest. The bars represent the standard error of the mean. The left panel represents females, and the right panel represents males.

To assess whether non-parametric methods could perform better than linear models by capturing potential interactions among genes, we fitted Random Forest models to the data. For female starvation resistance, Random Forest (*r* = 0.33 ± 0.01) had a greater prediction accuracy than TBLUP; whereas for male starvation resistance, the reverse was true: TBLUP had greater prediction accuracy than Random Forest (*r* = 0.31 ± 0.01) (Figure 3). For startle response, the prediction accuracies of TBLUP and the Random Forest model (*r* = 0.10 ± 0.01) were similar in females; but the prediction accuracy of the Random Forest model (*r* = 0.15 ± 0.01) was greater than TBLUP for male startle response (Fig. S5). Both models had no predictive ability for chill coma recovery in either sex (Fig. S6). Thus, there was no consistent difference in the performance of Random Forest compared to the TBLUP model.

### Transcriptome-Wide Association Study (TWAS) informed prediction

**Figure 4:**
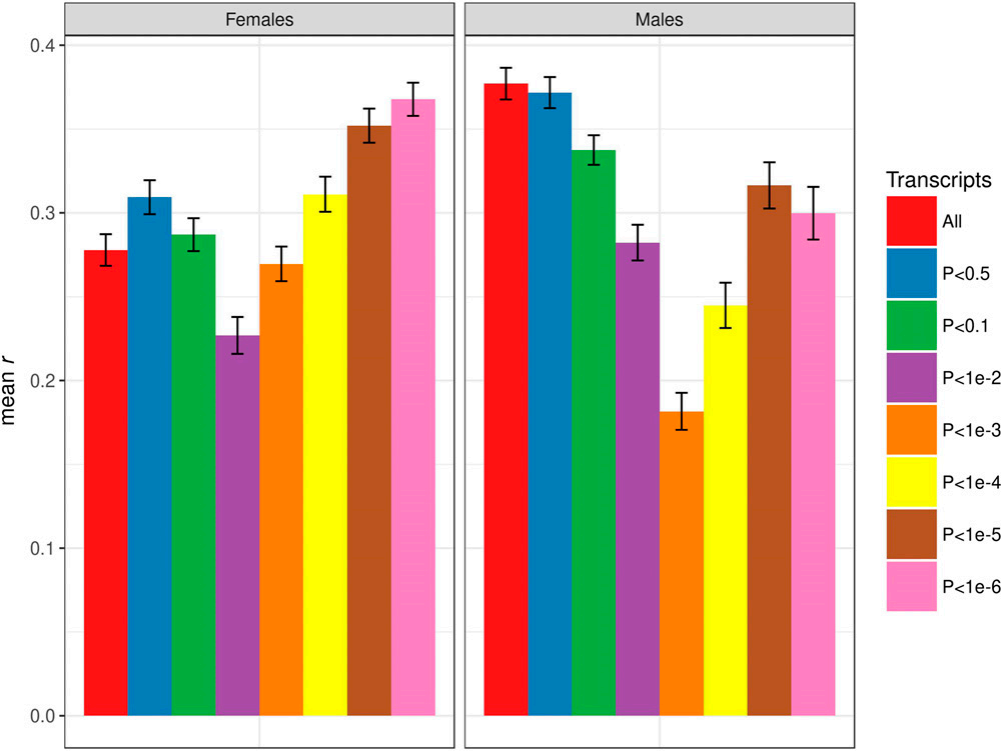
Prediction accuracy for starvation resistance, measured as mean *r* (on the y-axis) in the test set, obtained by the TWAS-TBLUP model at different *p*-value thresholds for selecting genes. The bars represent the standard error of the mean. The left panel represents females, and the right panel represents males.

We next combined mapping and prediction into a single pipeline in an attempt to enrich the TBLUP model for genes associated with the trait of interest (TWAS-TBLUP). For each trait and sex combination, we included transcripts associated with the trait in the training set with increasingly stringent *p*-vales (*P* < 0.5, 0.1, 10^−2^, 10^−3^, 10^−4^, 10^−5^, 10^−6^). Again, the results of these analyses varied by trait and sex. For female starvation resistance, the most stringent *p*-value threshold (10^−6^) significantly improved prediction accuracy (*r* = 0.37 ± 0.01) over using all highly expressed genes and gave the best accuracy overall. However, using all transcripts provided the highest prediction accuracy for male starvation resistance, where selecting transcripts always resulted in lower accuracy (Figure 4). For female startle response, the best prediction accuracy was for all transcripts; lowering the *p*-value threshold below 0.1 reduced the prediction accuracy to zero. For startle response in males, the best prediction accuracy was for *P* < 0.5 (*r* = 0.16 ± 0.01); lowering this threshold below *P* < 10^−2^ significantly decreased prediction accuracy (Fig. S7). Prediction accuracy for female chill coma recovery was again zero for all models; for male chill coma recovery, the model selecting genes with *P* < 10^−4^ gave the best, albeit very low, predictive ability (*r* = 0.07 ± 0.01) (Fig S8).

**Figure 5:**
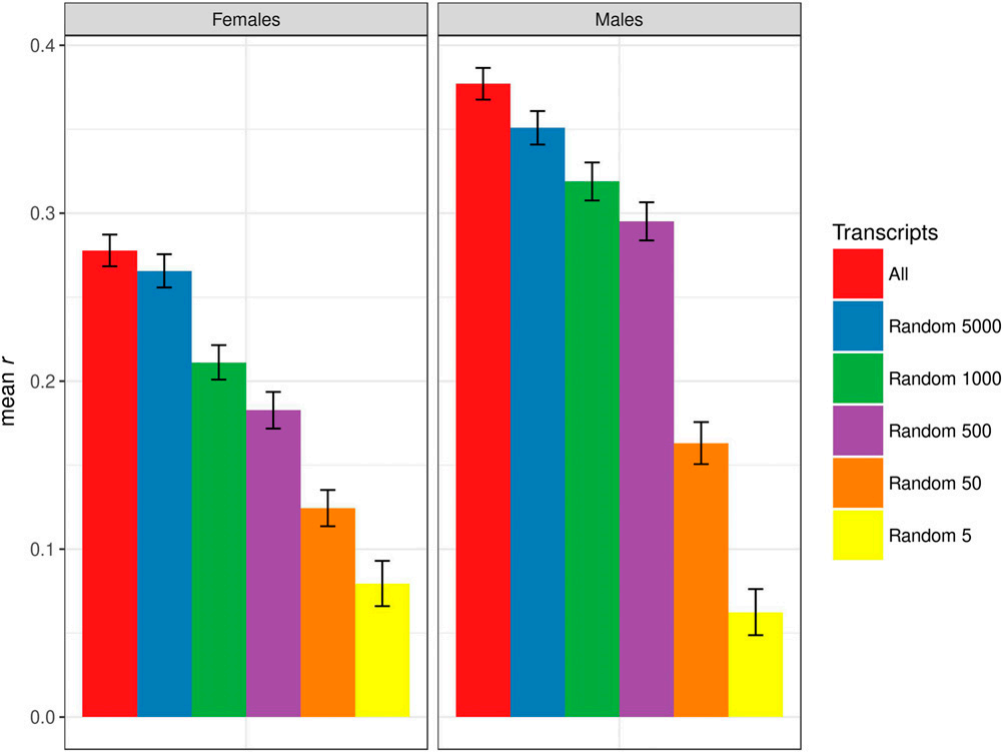
Prediction accuracy for starvation resistance, measured as mean *r* (on the y-axis) in the test set, obtained by using only different numbers of randomly selected genes in the TBLUP model. The bars represent the standard error of the mean. The left panel represents females, and the right panel represents males.

We also performed a control analysis in which we randomly selected 5000, 1000, 500, 50 and 5 transcripts with which to perform TBLUP prediction. For starvation resistance, decreasing the number of randomly selected genes monotonically decreased the prediction accuracy relative to the model including all transcripts, as expected (Figure 5). In particular, the prediction accuracies for 50 and 5 randomly selected genes for starvation resistance, corresponding roughly to the two highest *p*-value thresholds (*P* < 10^−5^ and *P* < 10^−6^), were reduced by two and fivefold, respectively, from the TWAS-TBLUP analysis (Figures 4, 5). However, 500 or more randomly sampled genes actually give significant prediction accuracies for starvation resistance in both sexes (Figure 5). This is also true for startle response, where 500 or more randomly sampled genes gave similar prediction accuracies to all transcripts in both sexes (Fig. S9). This is in contrast to the true TWAS-TBLUP analysis for this trait, where selecting genes with *p*-values for association with the trait of < 10^−2^ in females and < 10^−4^ in males gave very low to zero prediction accuracies (Fig. S7). Predictive abilities for chill coma recovery were zero or below for all models in females, and the randomly selected genes did not yield any positive prediction accuracies in male, in contrast to the true TWAS-TBLUP analysis, where significant prediction accuracies were found for transcript association *p*-values < 10^−4^ (Figs. S8, S10). We conclude from these results that the TWAS-TBLUP strategy was not effective for the three traits analyzed.

### Gene Ontology (GO) informed prediction

**Figure 6:**
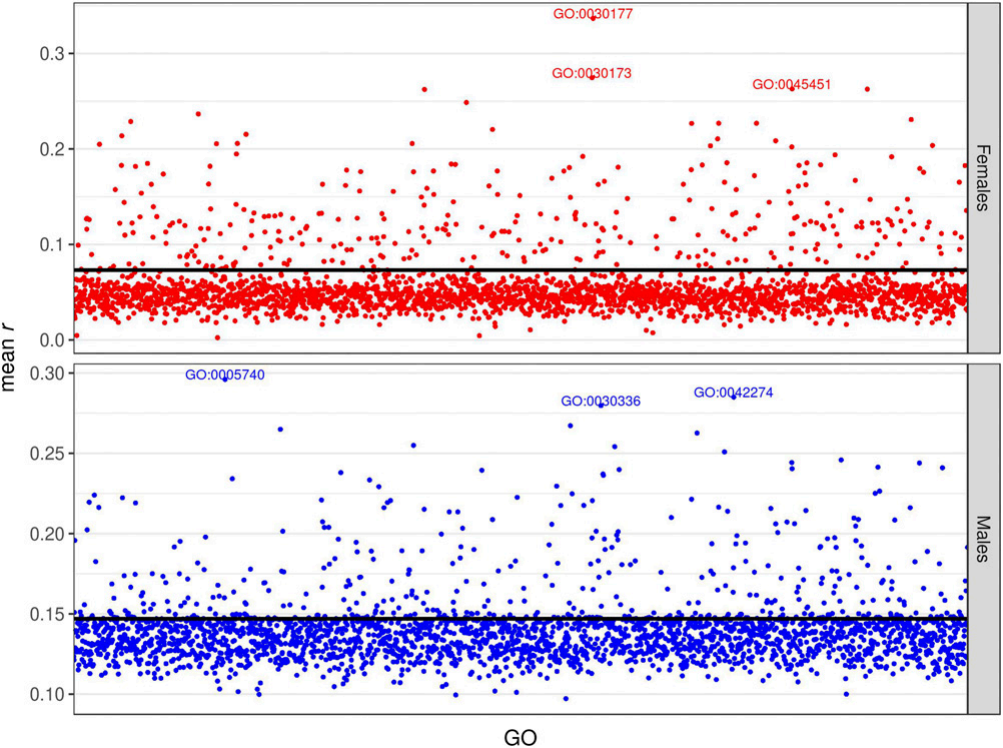
Prediction accuracy for starvation resistance, measured as mean *r* (on the y-axis) in the test set, obtained by the GO-GBLUP model. Each point represents the prediction accuracy achieved by a specific GO term; the top 3 most predictive GO terms are spelled out. The black horizontal line represents the accuracy of the baseline GBLUP model. The upper panel represents females, and the lower panel represents males.

We hypothesized that the lack of success of the TWAS-TBLUP model might have been due to the small size of the DGRP, providing low power to map genes (especially those with smaller effect) affecting the traits. We therefore assessed whether prediction accuracy could be improved by grouping variants and genes according to GO categories, and whether some GO terms are particularly predictive of the traits. First, we fitted the GO-GBLUP model proposed by Edwards *et al.* (2016). We found that the majority of GO terms provided similar accuracy to the baseline GBLUP model (the black horizontal line in the graphs), but some GO terms achieved much higher prediction accuracies for all the trait/sex combinations – even chill coma recovery time (Figures 6, S11, S12). Some of the most predictive GO terms had a clear interpretation (Table S1). The most predictive GO terms for starvation resistance in females affect development and reproduction and in males affect energy and protein synthesis. GO terms involved in translation elongation are predictive of chill coma recovery time, while GO terms involved in mitochondrial function are predictive of startle response (Table S1). Some of the top predictive GO terms for each trait are in common between the two sexes (GO:005741 and GO:007088 for startle response and GO:0033588 for chill coma recovery time), but most are different for males and females (Figures 6, S11, S12).

**Figure 7:**
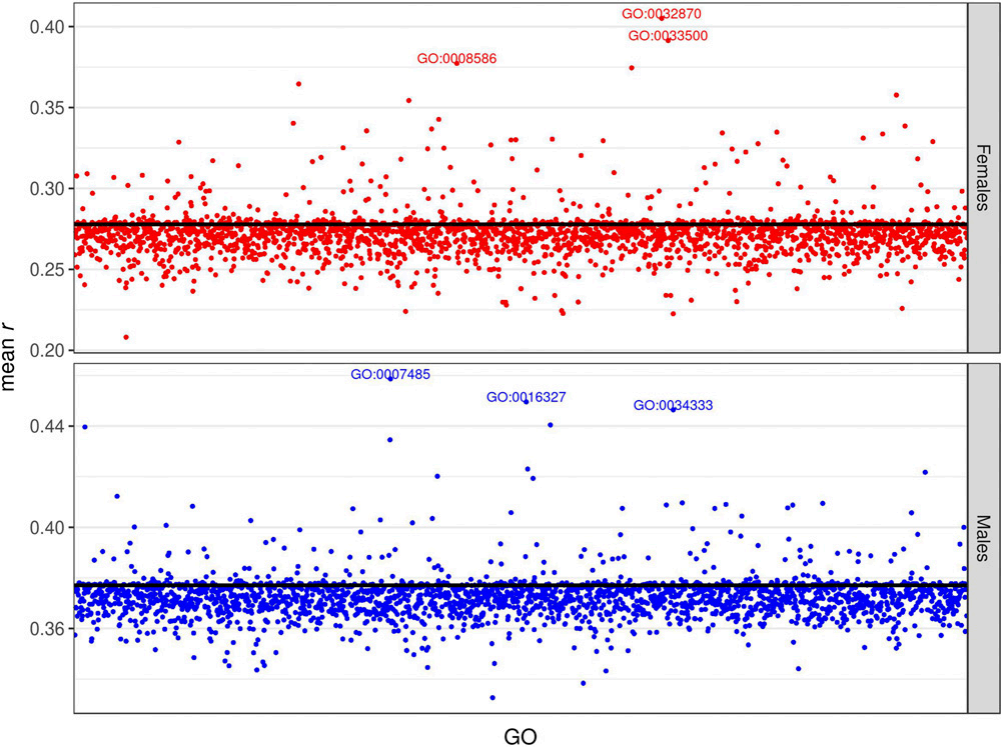
Prediction accuracy for starvation resistance, measured as mean *r* (on the y-axis) in the test set, obtained by the GO-TBLUP model. Each point represents the prediction accuracy achieved by a specific GO term; the top 3 most predictive GO terms are spelled out. The black horizontal line represents the accuracy of the baseline TBLUP model. The upper panel represents females, and the lower panel represents males.

We then developed the GO-TBLUP model and fitted it to our data. The results showed a very similar pattern to those of GO-GBLUP – most GO terms had very similar predictive abilities to TBLUP (the black horizontal line in the graphs), yet some GO terms provided much higher accuracies (Figures 7, S13, S14). Again, some of the most predictive GO terms had a clear interpretation. For example, two of the most predictive GO terms for starvation resistance in females, GO:0033500 and GO:0032870, have been implicated in carbohydrate homeostasis and cellular response to hormone stimulus (including the insulin receptor signaling pathway), respectively (Table S2) (Gramates *et al.* 2017). Several genes in these categories (*Sik2*, *Sik3*, *Akh*, *AkhR*) have previously been associated with starvation resistance (Table S2). In particular, *AkhR*, which is shared by both GO terms, was among the top associations with starvation resistance in both females and males in the DGRP (Everett *et al.* 2020). The three most predictive GO terms for male starvation resistance share *PDZ-GEF* (Table S2), which was also among the top associations with starvation resistance in males in the DGRP (Everett *et al.* 2020). The top predictive GO term for female startle response includes genes affecting chromatin organization (GO:0006325), while the top GO predictive GO terms for male startle response, GO:0030431 and GO:0006730, respectively affect sleep and one carbon metabolism (Table S2). Two of the most predictive GO terms (GO:0042052 and GO:0008103) for female startle response shared two genes, *Rab11* and *capulet*. Predictive GO terms affecting chill coma recovery include GO:0003755 in females (unfolded protein binding) and GO:0007266 and GO:0035025 in males (Rho signal transduction) (Table S2). None of the top predictive GO terms for the same trait are in common between males and females. Interestingly, the most predictive SNP-based GO terms and the most predictive transcript-based GO terms were distinct.

**Figure 8:**
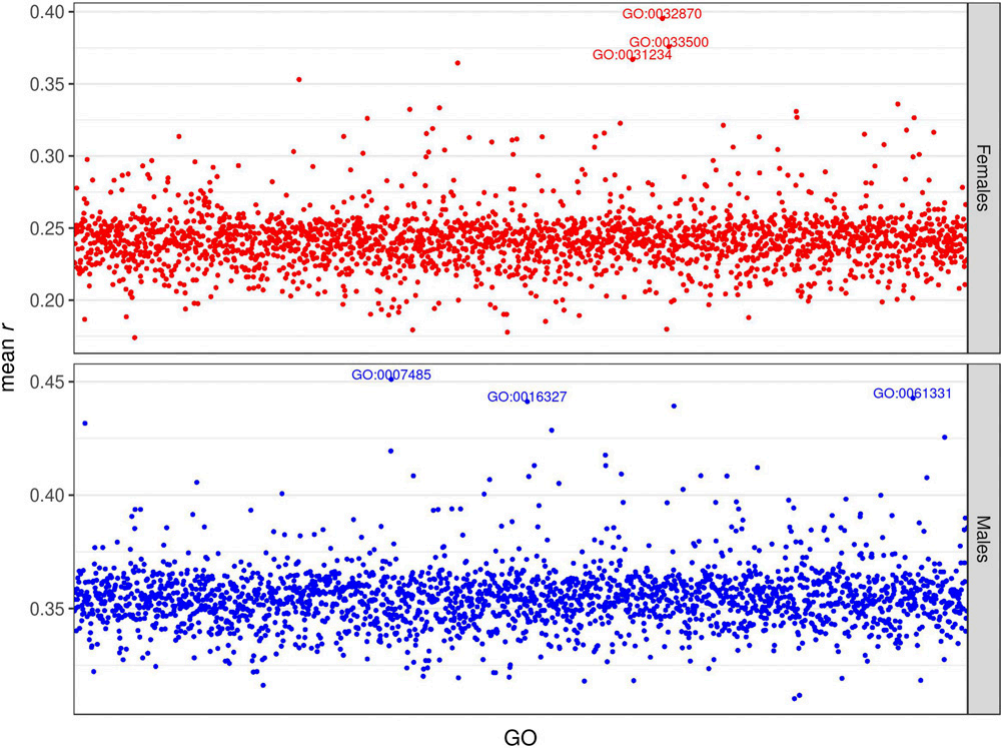
Prediction accuracy for starvation resistance, measured as mean *r* (on the y-axis) in the test set, obtained by the GO-GTBLUP model. Each point represents the prediction accuracy achieved by a specific GO term; the top 3 most predictive GO terms are spelled out. The upper panel represents females, and the lower panel represents males.

Finally, we fitted a GO-GTBLUP model to see whether genomic data and transcriptomic data contributed overlapping information to prediction accuracy for each GO. Generally, the combined model did not yield higher accuracy than the better of the GO-GBLUP and GO-TBLUP models; however, the ranking of the most predictive GO terms changes slightly (Figures 8, S15, S16). Only in one case, chill coma recovery in males, did this combined model achieve the best accuracy, although only marginally (Fig. S16).

**Figure 9:**
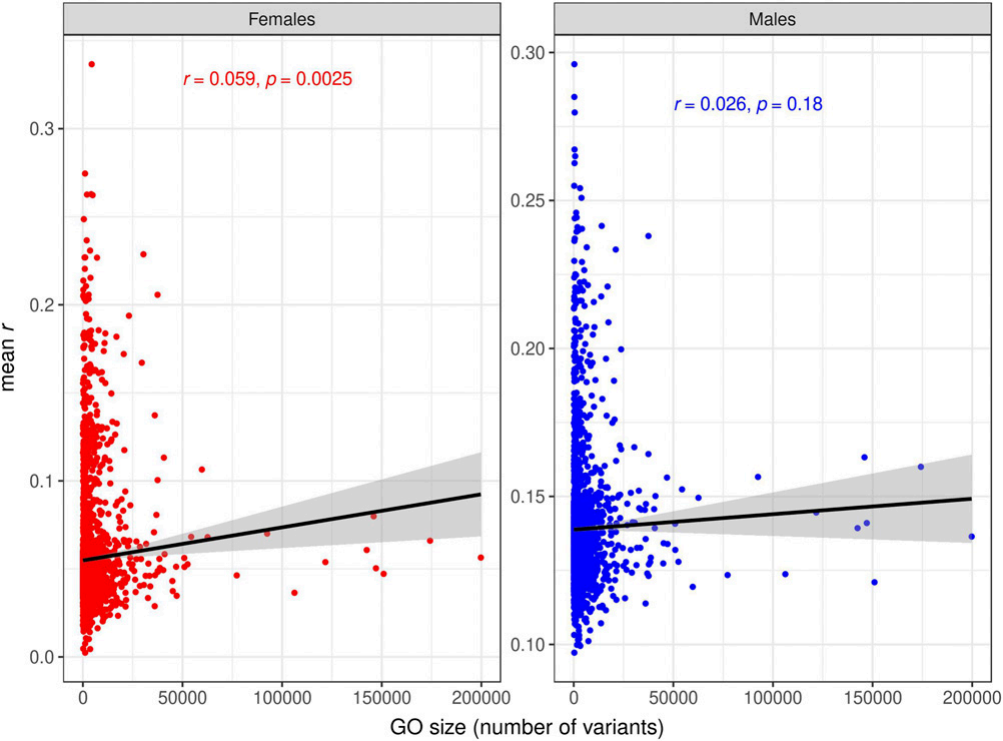
Relationship between prediction accuracy obtained by the GO-GBLUP model, measured as mean *r* (on the y-axis) in the test set, and size of the GO terms, measured as number of variants (on the x-axis), for starvation resistance. Each point represents a specific GO term. The black line represents the linear regression fit. The left panel represents females, and the right panel represents males.

**Figure 10:**
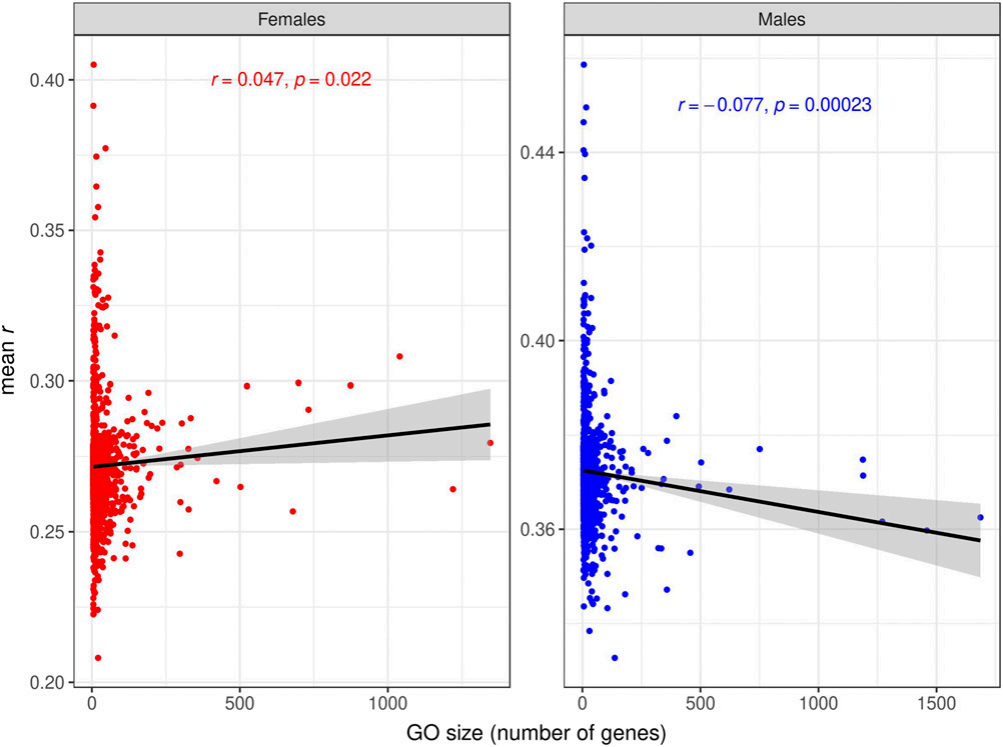
Relationship between prediction accuracy obtained by the GO-TBLUP model, measured as mean *r* (on the y-axis) in the test set, and size of the GO terms, measured as number of genes (on the x-axis), for starvation resistance. Each point represents a specific GO term. The black line represents the linear regression fit. The left panel represents females, and the right panel represents males.

**Figure 11:**
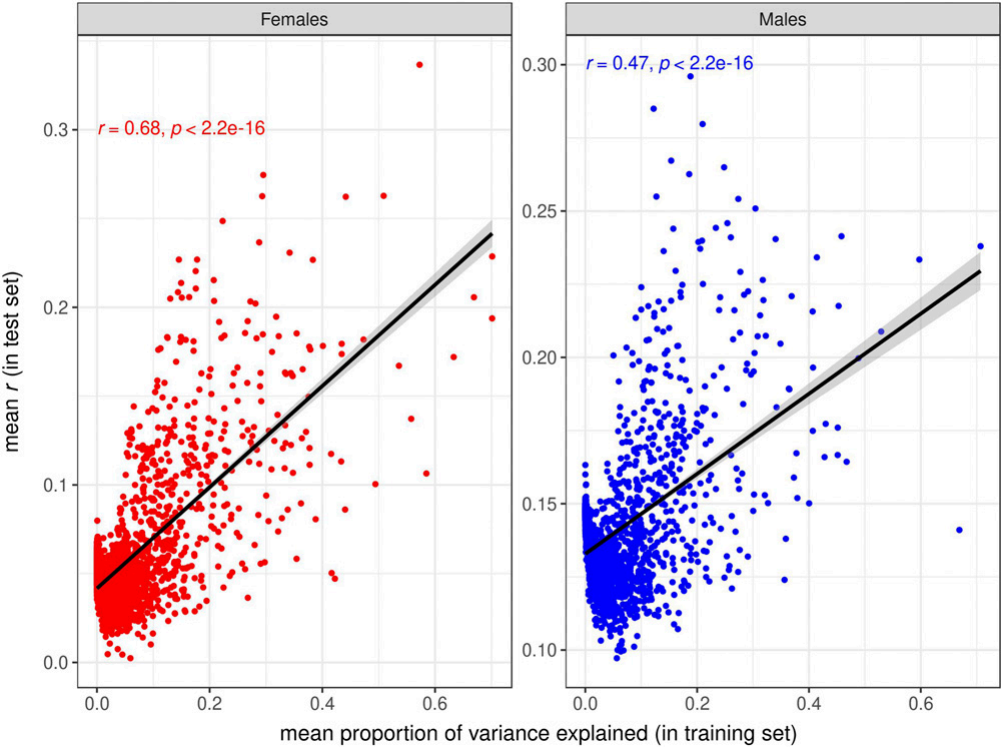
Relationship between prediction accuracy obtained by the GO-GBLUP model, measured as mean *r* in the test set (on the y-axis), and mean proportion of variance explained by the GO terms in the training set (on the x-axis), for starvation resistance. Each point represents a specific GO term. The black line represents the linear regression fit. The left panel represents females, and the right panel represents males.

**Figure 12:**
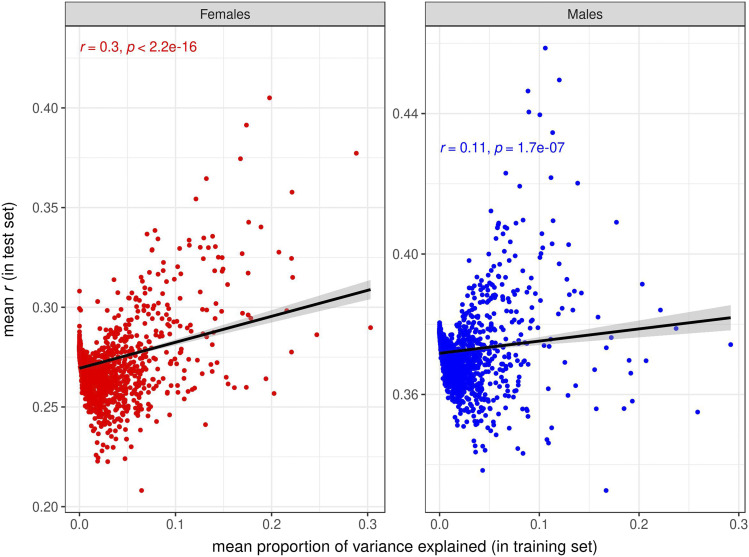
Relationship between prediction accuracy obtained by the GO-TBLUP model, measured as mean *r* in the test set (on the y-axis), and mean proportion of variance explained by the GO terms in the training set (on the x-axis), for starvation resistance. Each point represents a specific GO term. The black line represents the linear regression fit. The left panel represents females, and the right panel represents males.

Under an additive, infinitesimal model of genetic architecture in which loci affecting each trait have equal and infinitesimally small effects and are distributed evenly throughout the genome, a trivial reason for the increased prediction accuracy provided by some GO terms could be that those are simply the GO terms with larger number of variants/genes (Boyle *et al.* 2017). We tested this hypothesis, and found very low correlations between prediction accuracy and GO size for SNPs (Figure 9, Figs. S17, S18) and for transcripts (Figure 10, Figs S19. S20). In contrast, the correlations between prediction accuracies in the test set and proportion of variance explained in the training set were high for the GO-GBLUP (Figure 11, Figs. S21, S22) and GO-TBLUP (Figure 12, Figs S23, S24) models, as expected if the models are capturing biological signal rather than overfitting the noise.

## Discussion

Here, we evaluated the use of transcriptomic data for the prediction of complex traits, taking advantage of the unique resource of the DGRP, for which accurate mean values of organismal quantitative trait phenotypes (Huang *et al.* 2014; Mackay and Huang 2018) and genome wide transcript levels (Everett *et al.* 2020) have been obtained for the same population of genotypes. We used the genomic best linear unbiased prediction (GBLUP) model as a standard for comparison for transcriptome best linear unbiased prediction (TBLUP), as well as a combined genome and transcriptome model (GTBLUP) and the combined model including an interaction between the genome and transcriptome (GTIBLUP).

Using starvation stress resistance, startle-induced locomotion and chill coma recovery time as model quantitative traits (Huang *et al.* 2014), we first assessed the proportion of phenotypic variance explained for each model. We found that the proportion of genetic and transcriptome variance explained varied by trait and by sex within each trait. For starvation resistance, both the genome (GBLUP) and transcriptome (TBLUP) explained similar and high (∼70% in females, >90% in males) proportions of the phenotypic variance. The genome explained a higher proportion of variance for startle response than the transcriptome, especially in females; however, the total amount of variance explained for the genome and transcriptome was much lower than for starvation stress resistance in both sexes. While the transcriptome did not explain any of the phenotypic variance in chill coma recovery, the genome could explain a limited amount of variance in both sexes. The combined GTBLUP model did not explain a significantly larger proportion of variance than the better of the GBLUP or TBLUP models. The GTIBLUP model explained similar amounts of phenotypic variance for starvation resistance and startle response as the GTBLUP model. However, for chill coma recovery time, the GTIBLUP model explained ∼60% of the phenotypic variance for female chill coma recovery time (most of which came from the interaction term), tripling the best of the other models. These results indicate that the genome and transcriptome may contribute largely overlapping information, with the key player being dependent on the trait analyzed. This contrasts with previous results (Guo *et al.* 2016; Li *et al.* 2019; Azodi *et al.* 2020) in which the genome consistently explained more variance than the transcriptome in all models, and the interaction term contributed to explaining more variance for almost all of traits analyzed (Guo *et al.* 2016).

We next evaluated the performance of the four models in terms of prediction accuracy in a cross-validation setting. Prediction accuracy was low for starvation resistance and startle response, and zero for chill coma recovery, consistent with previous GBLUP results for these traits (Ober *et al.* 2012; 2015; Edwards *et al.* 2016; Li *et al.* 2019). One potential explanation for the null prediction accuracy in chill coma recovery could be the actual distribution of phenotypes not matching that assumed by mixed models (Ober *et al.* 2015). However, Ober *et al.* (2015) also fitted GBLUP to Box-Cox-transformed phenotypes and found no difference. This was also confirmed by the fact that a method that does not make any assumption regarding the distribution of the response variable – the Random Forest – provide similar (null) accuracy to TBLUP. There was a large gap between the proportion of variance explained and prediction accuracy for starvation resistance and startle response, also as previously observed (Morgante *et al.* 2018). Although the genome and transcriptome alone explained a similar and large amount of phenotypic variance for starvation resistance, prediction accuracies for the transcriptome out-performed the genome by 2.5 fold (males) to 3.5 fold (females). For startle response, the genome explained more phenotypic variance than the transcriptome in both sexes (much more for females); however, prediction accuracies were similar for the GBLUP and TBLUP models. Prediction accuracies for the GTBLUP and GTIBLUP models for any trait never exceeded those from the better of the single component models, bolstering the hypothesis that the genome and transcriptome contribute largely redundant information.

Our results comparing prediction accuracies of the different models agree with those of Guo *et al.* (2016) regarding the absence of an improvement in predictive ability when including the genome-transcriptome interaction term in the model. However, we did not observe an improvement in prediction accuracy for the GTBLUP model, in contrast to the previous study (Guo *et al.* 2016) which did find an improvement with the same combined model. This discrepancy may have occurred because we utilized only genes whose expression levels were genetically variable across lines in our transcriptome prediction models. This filter was necessary because the individual flies that were phenotyped were not the same flies from which RNA was extracted, although their genotypes were identical. Thus, genetics was the only link between these two sets of flies. Therefore, we will have missed contributions from any genes whose expression levels were not genetically variable but may have captured some (micro-) environmental effects. Our results also differed from those of a previous study (Li *et al.* 2019) using gene expression abundances derived from tiling arrays (Huang *et al.* 2015), which found TBLUP to be consistently much worse than GBLUP, providing null prediction accuracy for many traits. This is likely due to noisier expression measurements from tiling arrays compared to estimates of transcript abundance from RNA-seq (Everett *et al.* 2020) used in this study.

The GBLUP, TBLUP, GTBLUP and GTIBLUP models evaluated here all assume that all genomic variants and transcripts contribute equally to all traits; *i.e.*, the genetic architecture of each trait approaches the additive, infinitesimal model. However, single variant association mapping shows that the distribution of genetic effects for most quantitative traits follows a more exponential distribution, with some larger effect variants and increasingly more with increasingly smaller effects. This is why trait-specific variable selection models have greater predictive ability than GBLUP models including all variants, as the latter adds variants not associated with the trait (Ober *et al.* 2015; Morgante *et al.* 2018). However, when we applied this strategy to gene expression data using TBLUP, the results were very variable across traits, sexes and *p*-value thresholds. This might be attributable to the correlation structure among transcripts (Everett *et al.* 2020) differing for the traits and sexes with different *p*-value thresholds, but this is speculative and requires further investigation in the future.

The top associated variants typically can be mapped to known genetic or protein-protein interaction networks, or are enriched for plausible GO categories (Morozova *et al.* 2015; Carbone *et al.* 2016). Therefore, prediction methods based on mapping variants to genes and then to GO categories (genomic feature BLUP, Edwards *et al.* 2016; Rohde *et al.* 2017; 2018; Sørensen *et al.* 2017) can achieve high prediction accuracy than GBLUP. This is likely because these models can give higher weight in the prediction model to variants pertaining to biological processes or molecular functions specific to each trait (Edwards *et al.* 2016). Therefore, we assessed predictive ability of individual GO terms for each trait using the GO-GBLUP model (Edwards *et al.* 2016) as a baseline, and then extended this approach to GO-TBLUP. In agreement with Edwards *et al.* (2016), we found that a limited number of GO terms provided much higher accuracy with GO-GBLUP than the baseline GBLUP. The most predictive GO terms in this study differed from those of Edwards *et al.* (2016), which evaluated the same three quantitative traits in the DGRP, although the general ranking of the most significant GO terms was consistent between the two studies. Four factors likely contributed to these differences. (1) The two studies used different subsets of lines, which can give different quantitative outcomes with such a small sample size. (2) Our study used fivefold cross-validation as opposed to the 10-fold cross-validation employed by Edwards *et al.* (2016), and the size of the training set affects prediction accuracy (Ober *et al.* 2012). (3) We used line means for phenotypes, while Edwards *et al.* (2016) used individual measurements. (4) We used GO terms containing at least 5 genes while Edwards *et al.* (2016) used GO terms containing at least 10 genes.

The GO-TBLUP results also showed that a small number of GO terms achieved a much higher accuracy than the baseline TBLUP model, some of which shared genes that have been shown (statistically and/or functionally) to affect the traits analyzed. However, the most predictive GO terms in GO-GBLUP and the most predictive GO terms in GO-TBLUP were not the same. This suggests that while the genome and transcriptome as a whole may contribute redundant information, this is not true when gene ontology information is incorporated. This observation also suggests that it may be possible to build a trait-specific model with the most predictive SNP-based GO terms and the most predictive gene-based GO terms to improve the overall prediction accuracy. However, to be able to do so, it is necessary to develop a procedure to select the most predictive GO terms without bias in the training set, and more research is needed in that area.

Epistasis is a common hallmark of the genetic architecture of Drosophila quantitative traits (Huang *et al.* 2012; Shorter *et al.* 2015), and accounting for epistasis when it is present can improve genomic prediction (Ober *et al.* 2015; Morgante *et al.* 2018). The generally similar performance of TBLUP and Random Forest may suggest that gene expression affect these three traits mostly linearly. However, in a recent study, Random Forest showed a poor prediction performance with simulated phenotypes including a non-additive component (at the variant level) (Abdollahi-Arpanahi *et al.* 2020). Thus, further analyses are needed to elucidate this aspect. One such analysis could be the extension of GO-TBLUP (and GO-GBLUP) to account for epistatic interactions. This methodology would limit the inclusion of interactions at the GO level, which has both statistical (*i.e.*, fewer effects to estimate) and biological (*i.e.*, genes in GO categories often interact genetically) advantages.

In summary, this study has confirmed that using transcriptomic data to predict quantitative trait phenotypes is promising for some traits. Our work, together with other studies (Finucane *et al.* 2015; Edwards *et al.* 2016; Abdollahi-Arpanahi *et al.* 2017; Azodi *et al.* 2020), has shown that integrating omic data together with functional annotation can identify features that are important to understand and predict complex traits. However, there are several improvements to the experimental design that can be made in the future that may further increase predictive ability and consequently our understanding of the genetic basis of variation in quantitative traits. The most obvious improvement is to increase the sample size of the population on which organismal and omic phenotypes are assessed. The small size of the current DGRP limits the maximum prediction accuracy that can be achieved. Second, RNA was extracted from whole flies; this approach gives the average gene expression levels across all tissues. This may be advantageous, because identifying tissues relevant to specific traits is not trivial. However, it is conceivable that brain gene expression may be more relevant to predicting startle response than whole flies, and the other tissues that were not relevant to startle response added noise to the expression levels, potentially affecting prediction accuracy. Gene expression is known to be, at least partly, tissue-specific in both flies (Leader *et al.* 2018) and humans (Aguet *et al.* 2017). Third, RNA was extracted from flies that were reared in standard conditions and were not subjected to any external stimulus; however, all three traits analyzed were stress-based. This is exactly the situation to which precision medicine applies – predicting disease risk from baseline, healthy conditions. However, the transcriptome relevant to predicting a stress-related phenotype such as chill coma recovery time may well be a snap shot taken during chill coma or immediately following recovery rather than baseline, and might be one reason for the poor prediction accuracy of TBLUP for this trait. For starvation resistance, which had the highest prediction accuracy from TBLUP, expression levels on baseline flies may reflect their ability to store and mobilize energetic resources. This might explain the higher accuracy of TBLUP and agrees with the most predictive GO term being implicated in carbohydrate homeostasis. Finally, the GO-GBLUP and GO-TBLUP models are very flexible and can be extended in the future to incorporate other omic levels as well as regulatory features located outside of coding regions.
